# Macro-, Micro- and Nano-Roughness of Carbon-Based Interface with the Living Cells: Towards a Versatile Bio-Sensing Platform

**DOI:** 10.3390/s20185028

**Published:** 2020-09-04

**Authors:** Lena Golubewa, Hamza Rehman, Tatsiana Kulahava, Renata Karpicz, Marian Baah, Tommy Kaplas, Ali Shah, Sergei Malykhin, Alexander Obraztsov, Danielis Rutkauskas, Marija Jankunec, Ieva Matulaitienė, Algirdas Selskis, Andrei Denisov, Yuri Svirko, Polina Kuzhir

**Affiliations:** 1 Center for Physical Sciences and Technology, Sauletekio Ave. 3, LT-10257 Vilnius, Lithuania; lena.golubewa@ftmc.lt (L.G.); renata.karpicz@ftmc.lt (R.K.); danielis@ar.fi.lt (D.R.); ieva.matulaitiene@ftmc.lt (I.M.); algirdas.selskis@ftmc.lt (A.S.); 2 Institute for Nuclear Problems, Belarusian State University, Bobruiskaya 11, 220030 Minsk, Belarus; tatyana_kulagova@tut.by; 3 Institute of Photonics, University of Eastern Finland, Yliopistokatu 2, FI-80100 Joensuu, Finland; hamzar@uef.fi (H.R.); marian.baah@uef.fi (M.B.); tommi.kaplas@gmail.com (T.K.); sergeim@uef.fi (S.M.); alexander.obraztsov@uef.fi (A.O.); yuri.svirko@uef.fi (Y.S.); 4 Department of Biophysics, Belarusian State University, Nezavisimosti Ave. 4, 220030 Minsk, Belarus; denisov@bsu.by; 5 Department of Micro and Nanosciences, Aalto University, FI-00076 Espoo, P.O. Box 13500, Finland; ali.shah07@outlook.com; 6 Division of Solid State Physics, Lebedev Physical Institute of the Russian Academy of Sciences, Leninskiy Prospekt 53, 119991 Moscow, Russia; 7 Department of Physics, Lomonosov Moscow State University, Leninskie gory 1–2, 119991 Moscow, Russia; 8 Institute of Biochemistry, Life Sciences Center, Vilnius University, Sauletekio Ave. 7, LT-10257 Vilnius, Lithuania; marija.jankunec@gmc.vu.lt; 9 Institute of Physiology of the National Academy of Sciences of Belarus, Minsk, Belarus, 28 Akademichnaya Str., BY-220072 Minsk, Belarus

**Keywords:** pyrolytic carbon, graphene, graphene nanowalls, black silicon, glioma cells, biocompatibility

## Abstract

Integration of living cells with nonbiological surfaces (substrates) of sensors, scaffolds, and implants implies severe restrictions on the interface quality and properties, which broadly cover all elements of the interaction between the living and artificial systems (materials, surface modifications, drug-eluting coatings, etc.). Substrate materials must support cellular viability, preserve sterility, and at the same time allow real-time analysis and control of cellular activity. We have compared new substrates based on graphene and pyrolytic carbon (PyC) for the cultivation of living cells. These are PyC films of nanometer thickness deposited on SiO_2_ and black silicon and graphene nanowall films composed of graphene flakes oriented perpendicular to the Si substrate. The structure, morphology, and interface properties of these substrates are analyzed in terms of their biocompatibility. The PyC demonstrates interface biocompatibility, promising for controlling cell proliferation and directional intercellular contact formation while as-grown graphene walls possess high hydrophobicity and poor biocompatibility. By performing experiments with C6 glioma cells we discovered that PyC is a cell-friendly coating that can be used without poly-l-lysine or other biopolymers for controlling cell adhesion. Thus, the opportunity to easily control the physical/chemical properties and nanotopography makes the PyC films a perfect candidate for the development of biosensors and 3D bioscaffolds.

## 1. Introduction

The importance of cell–surface contact for the living cell functioning has been known for decades. For example, for granulocytes, surface contact regulates the production of superoxide which is one of the milestones in immune response [1]. Similarly, neutrophils recruitment for the host defense via the inflammatory response is also initiated by cell adhesion and eventual migration through the vascular wall [2], and platelet activation and aggregation during blood vessel injury in the arterial circuit is triggered through the interaction of immobilized vWF with GPIb receptors after adhesion [3]. Extrinsic signals from the stem cell niche regulate neural stem/progenitor cells self-renewal [4] and the time course of neural stem cell fate determination [5]. Binding of the cell to the extracellular matrix and other cells connecting them with the surroundings play a crucial role in stem cell development including proliferation, differentiation, and migration, involved in organism repair processes. Thus, tuning the cell–surface interaction has tremendous potential in treating various disorders and injuries.

Cell–surface interactions and signaling events are two closely related processes. Among the main characteristics of the substrate responsible for its interaction with cells are (i) physical properties (stiffness, viscoelasticity, porosity, etc.), (ii) morphology (scaffold dimensionality (2D or 3D); thickness; area, shape, microscale topography of cell adhesion surface and nanotopography), and (iii) biochemical characteristics (affinity, the specificity of epitope interaction with cell receptors, nonaffinity domains, supramolecular organization, etc.) [6].

All these factors should be taken into account when integrating new materials in biosensors, scaffolds, implants, etc. toward cell or tissue regulation and engineering. Unfavorable reactions due to tissue scar [7] and unstable mechanical and electrical properties of silicon, which is conventionally used in the implants for the monitoring and regeneration of neurons, for instance, fuel the search for a viable alternative among both newly developed and traditional materials. The key requirements for the material to be met are biocompatibility, the possibility of using them as scaffolds for 3D cell growth (for tissue reconstruction), and the potential for biosensing platforms. Finding such materials, which should provide ample opportunities for the development of effective biocompatible sensing systems for monitoring cell activity, implies an analysis of surface properties and revealing the correlation between specific surface parameters and cell functional state.

Along with self-assembled monolayers and covalently bound peptides [8,9], carbon nanomaterials are being considered as a very promising candidate in the cultivation of living cells [10,11,12] and tissue engineering. Their advantageous—for bio-engineering—properties are the following: (i) they do not trigger unwanted reactions being injected into the tissue, (ii) they prevent blood clotting and exhibit good durability, (iii) they are robust and wear-resistant [13]. For instance, carbon nanotube-layered substrates are biocompatible and efficient in inducing stem cell differentiation, specifically to neurons [14], promoting neurite outgrowth and enhancing synaptogenesis [15] and consequential development of neuronal network [16].

Graphene promotes the growth and differentiation of stem cells into neurons offering a very attractive strategy for the treatment of neuronal injuries [17]. It has been also found that coating surfaces with graphene results in the acceleration—in comparison with bare surfaces—of stem cell growth [18]. Pristine graphene offers better electrical conductivity and further enhances the electric field of the cell; therefore, it is promising for cell culture and regeneration [19].

Three-dimensional (3D) graphene foam synthesized by chemical vapor deposition (CVD), which is biocompatible and shows no toxicity whatsoever, can be used as a conductive scaffold for neural stem cells [20]. The ripples and wrinkles on the surface of 3D graphene foams facilitate better interlocking of the cell with substrate stimulating proliferation and assisting the neural cell differentiation.

It is important to ensure the growth and functioning of cells on such surfaces of sensors. This biocompatible approach allows fulfilling the effective pharmacological screening and the modeling of various forms of various diseases in vitro, such as ischemia, trauma, stroke, epilepsy, Alzheimer’s disease, cancer, etc. [21]. On the other hand, microelectrode sensors are used for implantation into the structures of the central nervous system for registration and modulation of physiological processes in vivo. When electrodes are implanted into the brain, an acute reaction is initially observed at the point of contact between the brain tissue and the microelectrode material [22].

Our study was performed with astrocyte-like glioma cells. We concentrate on the creation of the biointerface of carbon nanomaterials and glioma cells to analyze the possibility of directed cell growth without using poly-l-lysine or other biopolymers for controlling cell adhesion. In this paper, we report on the biocompatibility of the surfaces based on graphene nanowalls (GNW) [23] and pyrolytic carbon (PyC) [24]. The GNW film comprises self-assembled graphene flakes with few nanometers thicknesses, which are vertically oriented to the substrate and have the lateral size of a few microns [25]. The PyC film is composed of graphene flakes predominantly parallel to the substrate, which have the lateral size of a few nanometers and are embedded into the amorphous carbon matrix [26].

PyC implants have been already used in human bodies suffering from arthritis, and no side effects have been experienced by the trial subjects [27]. Properties of PyC mostly depend on its synthesis conditions [28]. The combination of prominent electrical properties with relatively good optical characteristics [24], high thermal conductivity [29], and high chemical wear resistance and durability [30] make PyC film a promising coating material for a very broad spectrum of applications, including optical and electrical sensors, lab-on-a-chip schemes, scaffolds, implants, and almost in any device that must function stably in biological environments. Successful efforts have been already made in the creation of patterned PyC and tetrahedral amorphous carbon thin films for biocompatible surfaces [31]. Nevertheless, the question of the effect of surface roughness, as well as its nano and microstructuring on the adhesion of cells to the surface and the rate of their division, remains poorly understood.

Here, we investigated the influence of carbon synthesis conditions and the template/substrate properties on the final bioscaffold biocompatibility. By using different configurations of carbon-based interfaces including GNW on Si wafer, PyC on SiO_2_, and PyC-coated black silicon (bSi) we demonstrate that biocompatibility is strongly influenced by the substrate morphology. We show how the micro- and nanostructuring of carbon substrate affects the cell growth and adhesion and prove that the graphene and PyC can control the cell growth and adhesion.

## 2. Materials and Methods

### 2.1. Substrate Synthesis

#### 2.1.1. Black Silicon (bSi)

BSi refers to silicon surfaces covered by a layer of “needle” or “pyramidal”-like nano- or microstructures that suppress reflection and enhance the scattering and absorption of light [32,33]. The details of bSi fabrication can be found elsewhere [34]. Briefly, the fabrication of bSi was carried out with an Oxford Instruments Plasmalab system 100-ICP180 which has a double plasma source that allows for the independent control over density and directionality of ions. The plasma is created at a pressure of 13.3 Pa within the temperature range from −150 to 400 °C. Tall bSi pyramid-like structures were obtained at a pressure of 10 mTorr with an SF6 flow rate of 40 sccm, O_2_ flow of 18 sccm at −110 °C for 7 min, followed by 7 min of etching.

#### 2.1.2. PyC on SiO_2_ and PyC on bSi

PyC deposition on various surfaces might have some limitations, because the coefficient of thermal expansion (CTE) of the coating and the substrate must be similar. Otherwise high-temperature deposition of PyC on the substrate with the too different CTE from that of PyC will lead to dimensional interferences at room temperature and stresses generation, which will compromise PyC adherence [35]. However, the deposition of PyC on bSi and SiO_2_ overcomes this problem as CTE for Si, SiO_2_, and PyC is 0.54 × 10^−6^ K^−1^, 0.55 × 10^−6^ K^−1,^ and 0.29 × 10^−6^ K^−1^ [36], respectively. Moreover, direct deposition and PyC films of different thickness stability were demonstrated in [24].

For this particular study, the PyC films were deposited via CVD on the SiO_2_ or bSi substrates. The CVD chamber was sealed with the sample substrate inside, and H_2_ was injected for 2 h at a flow rate of 20 sccm and 100 Pa pressure to clean the substrates. Next, the pumping out was stopped and by flowing the H_2_, the chamber pressure was increased to approximately 1 kPa. Then, the H_2_ flow was stopped, and the CVD chamber was heated to 700 °C in a static H_2_ environment. With H_2_ already inside, CH_4_ was injected into the chamber at 30 and 50 sccm, allowing the chamber pressure to reach approximately 2.44 kPa. The chamber was further heated to 1100 °C at a rate of 10 °C/min. During the procedure, there was no active flow of gasses and the entire process was performed in static conditions. After resting for 5 min, the chamber was cooled down to 700 °C at a rate of 5 °C/min. Then, the CH_4_-H_2_ mixture was pumped out, replaced with H_2_ (20 sccm) at 1 kPa, and allowed to cool overnight. The dependence of the PyC film thickness on CVD synthesis conditions (CH_4_ to H_2_ concentration ratio, time, and temperature of synthesis) was specified in [26].

The optical properties of PyC films were previously investigated, and major features and characteristics of PyC are reported in [28]. In particular, it was shown that the dielectric permittivity of the PyC films deposited on the silica substrate is quite similar to those obtained for graphite and graphene, allowing the broadcasting of all applications of graphene as a material for optical biosensors on PyC films.

#### 2.1.3. Graphene Nanowalls (GNW)

The GNW films were deposited on a silicon substrate by a plasma-assisted CVD process. CH_4_–H_2_ mixture with a methane concentration of about 6.5% was activated by DC discharge. The deposition process was carried out within the temperature range from 850 to 1100 °C at 9.6 kPa for about 45 min [37]. In the deposited films, the multilayer graphene flakes of nanometer thickness with atomic layers oriented predominantly along a normal to Si substrate surface, while their lateral size (along substrate surface) and height (along normal to the substrate surface) are of a few microns. Both EDX and electrochemical analysis of graphene nanowalls carried out in [38] did not reveal the presence of any elements other than C (nanowalls themselves), Si (substrate), and O (oxygen on the sample due to exposure to air). The amount of impurities was 0.05–0.06 wt.%, which is less than the minimum detectable by an EDX value of 2000 ppm.

### 2.2. Structural and Morphological Characterization

#### 2.2.1. Scanning Electron Microscopy (SEM)

SEM micrographs of investigated samples were obtained by a Helios NanoLab 650 microscope (FEI, Netherlands, 2011) with a Schottky type field emission electron source. The cathode current was 25 pA at the tilt angle of 57°. The PyC on SiO_2_ samples was coated with chromium to improve the image quality and prevent the overcharging on the surface. The thickness of the PyC layer on SiO_2_ and bSi was determined from the lateral cleavage of the sample from SEM images. PyC has a clearly visible interface with a semiconductor/dielectric. Using the built-in SEM software, the thickness of the PyC film was determined at 10 different points, and then the mean value and standard deviation were calculated.

#### 2.2.2. Atomic Force Microscopy (AFM)

AFM imaging was carried out in a tapping mode on a Dimension Icon (Bruker, Santa Barbara, CA, USA) scanning probe microscope system at room temperature (20 ± 0.5 °C) using silicon AFM probes Multi75AI-G (BudgetSensors, Sofia, Bulgaria), for relatively rough surfaces of bSi with PyC and bSi, and Tap300AI-G (BudgetSensors, Sofia, Bulgaria) for smoother surfaces of PyC on SiO_2_. The scans were performed at a 512 pixel or higher resolution with a scan rate of 0.3–0.5 Hz. The imaged area was 2 × 2 µm. AFM measurements were carried out in at least three replicates.

Roughness parameters were calculated using the Build-In Software NanoScope Analysis version 1.90 (Bruker). According to the NanoScope Analysis 1.90 Manual, the Image R_q_ is defined as the Root mean square average of height deviations taken from the mean image data plane and can be presented in the following form:

(1) $$R_{q}=\sqrt{\frac{\sum Z_{i}{}^{2}}{N}}$$

Image R_max_ is the maximum vertical distance between the highest and lowest data points in the image following the planefit. Image Surface Area Difference is the difference between the surface area of the substrate and the area of its projection on the plane.

#### 2.2.3. Raman Spectroscopy

Raman measurements were performed using a Raman spectrometer/microscope inVia (Renishaw, UK). Spectra were analyzed with the custom WIRE software. A 785 nm near-infrared (NIR) laser source was used for all the samples. Objective magnification was 50×. The grating was with 1200 grooves/mm. The power at the sample was adjusted to 1 mW. The exposure time was 10 s, and the spectra were accumulated 3 times in one point to obtain a better signal-to-noise ratio. At least three different points were measured for each sample. The intensity units were counts per second.

### 2.3. Cell Culture Growth and Visualization

#### 2.3.1. Cell Culture

ATCC C6 (ATCC^®^ CCL-107™) rat glioma cells, obtained from ATCC, LGC Standards, Ogrodowa 27/29, Kielpin, Poland, were used in all experiments. Cells were grown in DMEM/F12 medium (Gibco, Grand Island, NY, USA) supplemented with 10% fetal bovine serum (Sigma Chemical Co., St. Louis, MO, USA) and 80 µg/mL gentamicin sulfate (Belmedpreparaty, Belarus) in 100% humidity at 37 °C in 5% CO_2_ atmosphere. In the log-phase cells were trypsinized, centrifuged, and 10^5^ cells were seeded in standard 3.5 cm plastic Standard Nunc™ Cell Culture Petri dishes with investigated substrates (bSi, bSi with PyC, SiO_2_-PyC (2 types) and GNW) placed on the Petri dish bottom. None of the sample surfaces were treated with poly-l-lysine for additional improvement of cell adhesion to the substrate. Cells were cultured for 3 days. For the measurements, the growth medium was replaced with Hepes buffer solution (pH 7.2) of the following content (in mmol/L): NaCl—131, KCl—5, MgSO_4_—1.3, CaCl_2_—1.3, Hepes—20, C_6_H_12_O_6_—5.

#### 2.3.2. Fluorescence Microscopy

Cell visualization was performed using DNA-staining fluorescent dye propidium iodide (Sigma-Aldrich, Germany). Before each measurement, substrates with cells on them were washed twice with Hepes buffer solution, fixed with ice-cold methanol for 30 min at 4 °C. Then substrates with cells were washed twice in Hepes buffer solution for 10 min each time and once with deionized water. During the measurements, water was replaced with 10^−6^ mol/L propidium iodide solution in Hepes buffer. The excitation wavelength was 532 nm and the emission maximum was 617 nm. The imaged area was 200 × 200 µm.

#### 2.3.3. Data Analysis

Measurements of average surface area, occupied by cells during their growth on different substrates (confluence), as well as morphological features analysis were performed using specific built-in functions of the open-source software ImageJ (version 1.51J8). For confluence determination, 7–10 fluorescent images (200 × 200 µm) were analyzed.

The results are expressed as the means ± standard deviations or means ± confidence intervals. Statistical analysis for 7–10 independent samples was conducted using the one-way ANOVA (*p* < 0.05); the statistical significance of the results was analyzed by an unpaired two-tailed Student’s *t*-test using Microsoft Office Excel 2010 (Microsoft Corporation, Redmond, Washington, DC, USA) and STATISTICA 6.0 (StatSoft Inc., Tulsa, Oklahoma, OK, USA). Statistical significance is indicated in the table footnote.

## 3. Results

**Figure 1:**
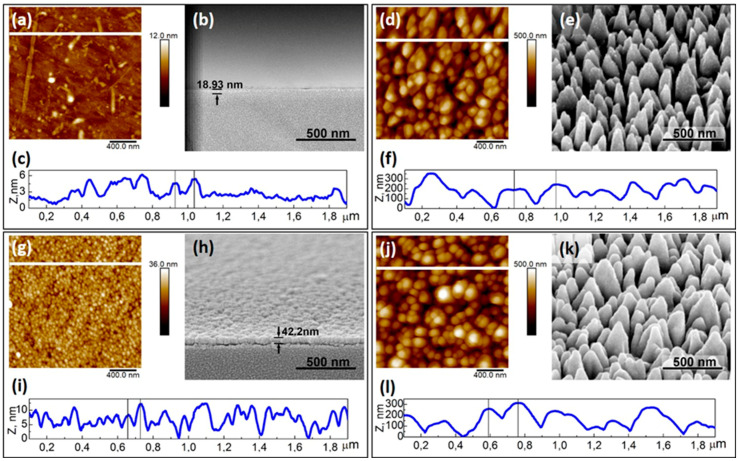
Typical two-dimensional AFM and SEM surface images of PyC of 20 nm (**a**,**b**) and 40 nm thickness (**g**,**h**) on SiO_2_, bSi (**d**,**e**), PyC of 20 nm on bSi (**j**,**k**), respectively. PyC layer is marked with arrows (**b**,**h**); (**c**,**f**,**i**,**l**) cross-sectional profiles of PyC (20 nm), bSi, PyC (40 nm), PyC (20 nm) on bSi from (**a**,**d**,**g**,**j**) AFM images, respectively. The cross-sectional profiles are marked with a white line on the AFM images (**a**,**d**,**g**,**j**).

To characterize the fabricated samples, we employed AFM and SEM imaging (Figure 1). The statistical results of AFM data analysis are summarized in Table 1.

Figure 1b,h show the SEM images of the PyC films of (42 ± 11) nm and (19 ± 7) nm thickness, respectively, deposited on SiO_2_. For simplicity, we dubbed samples “PyC (40 nm)” and “PyC (20 nm)”, respectively. One can observe that both films are uniform over the substrate, even though from the comparison of the AFM images in Figure 1a,c, it appears that the 40 nm thick film exhibits a grainy structure. The analysis of the AFM data summarized in Table 1 shows that the 20 nm thick PyC film (R_q_ = 0.9 ± 0.2 nm) was much smoother than the 40 nm thick PyC film (R_q_ = 2.9 ± 1.3 nm). This can be seen from Figure 1i,c which show the thicknesses of the film as a function of the lateral coordinate. It is worth noting that the 40 nm thick film was much denser than its 20 nm counterpart, which had rather scarce high amplitude peaks.

BSi consisted of pyramid-like silicon structures that were randomly distributed through the substrate surface as shown in Figure 1d,e. Figure 1j,k show the bSi coated with a 20 nm thick PyC film. One can observe from Figure 1e,k that a pyramid-like surface structure of the bSi endured PyC film deposition, even though the apex curvature of the pyramids increased resulting in a much smoother surface. Such a smoothing of the bSi after deposition of the 20 nm thick PyC film is quantitatively described by the decrease in the roughness from 81 ± 12 nm to 75 ± 9 nm (see Table 1). The difference between the maximum and minimum points of the tip position R_max_ also increased after depositing the PyC film. Nevertheless, statistical analysis of two independent datasets (bSi and bSi with PyC) does not allow us to suggest that differences between these datasets are statistically significant.

**Figure 2:**
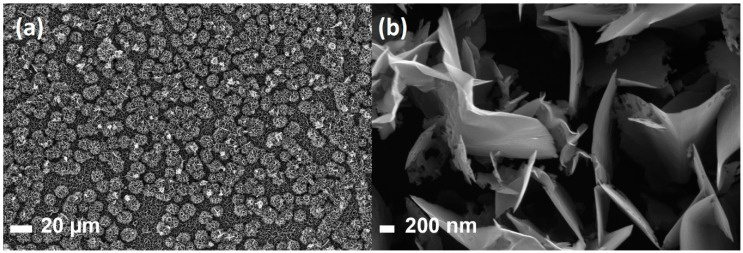
SEM images of graphene nanowalls; (**b**) the enlarged image of the area of “bushes” made of graphene sheets in (**a**).

In contrast to the PyC, the nanoscale roughness of the GNW film, which consisted of graphene flakes oriented perpendicular to the silicon substrate, was suppressed. In the SEM image of the GNW film surface presented in Figure 2, one can observe that GNWs formed “bushes” of 20 µm in diameter. They were distributed fairly evenly over the entire surface of the substrate (see Figure 2a) and were composed of vertically oriented multilayer graphene flakes (Figure 2b).

**Figure 3:**
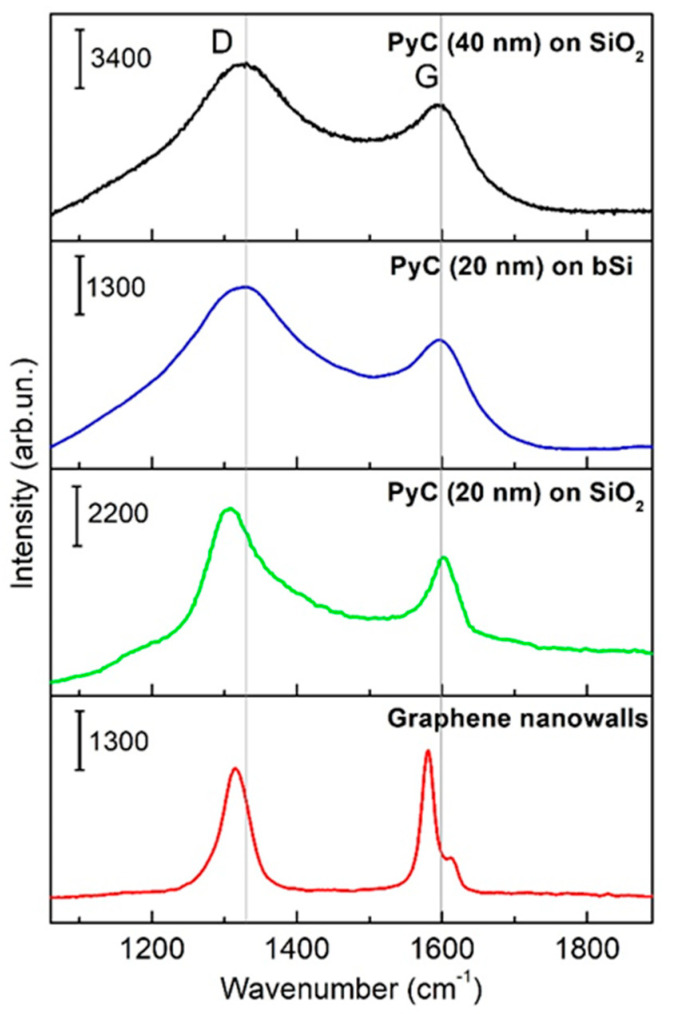
Raman spectra of PyC (40 nm) on SiO_2_, PyC (20 nm) on SiO_2_, PyC (20 nm) on bSi, and graphene nanowalls. λ_ex_ = 785 nm. Presented spectra are average spectra from triplicates. All scales are marked with corresponding scale bars on each individual spectrum.

Raman spectra of the PyC and GNW films are presented in Figure 3, while positions of the D and G peaks are summarized in Table 2. The Raman peaks of the 40 nm thick PyC film were approximately 7 cm^−1^ red-shifted to those of the 20 nm thick PyC film due to an increase in the internal stress in the thicker film [39]. This is because the composition of the pyrolytic carbon film does not depend on its thickness [40]: it comprises the graphene flakes embedded into the amorphous graphene matrix. Since the chemical conditions for the synthesis of the PyC films of different thicknesses were equivalent, the difference in the positions of the peaks caused by surface functionalization, which could occur during the synthesis process, was not expected. Following the theoretical estimations [41], peak displacement is most likely a result of mechanical stresses. Such a conclusion is supported by the fact that there was no shift in the position of the G-mode in the Raman spectrum of the PyC deposited onto SiO_2_ and bSi.

**Figure 4:**
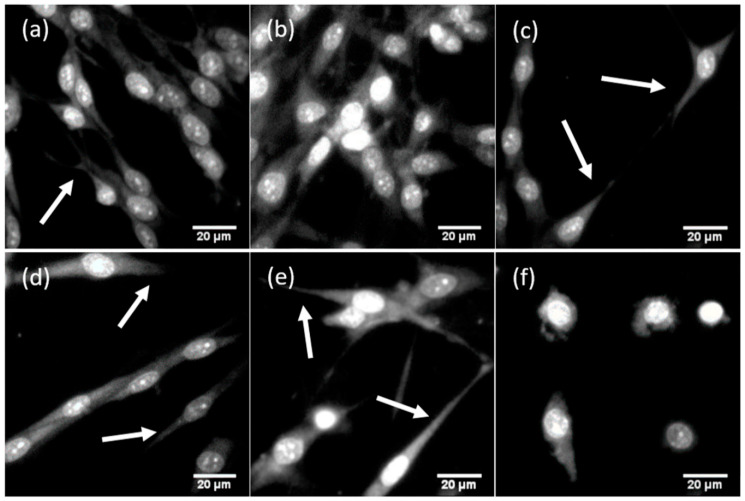
Typical fluorescent images of glioma cells grown on substrates with different roughnesses. Glioma cells were grown on (**a**) plastic (as a control); (**b**) bSi; (**c**) PyC (20 nm) on bSi; (**d**) PyC (20 nm) on SiO_2_; (**e**) PyC (40 nm) on SiO_2_; (**f**) graphene nanowalls. Long processes are marked with arrows. Cells were stained with propidium iodide. Image size 115 × 115 µm.

Affinity and biocompatibility of the fabricated substrates to living cells were analyzed in terms of average area occupied by cells in culture (confluence), average cell number, the average area per one cell, and morphological features of cells grown on the substrates. The results of the cell morphology analysis on fluorescent images of glioma cells fixed on the substrates (Figure 4) are summarized in Table 3. One can observe from Figure 4a,b that bSi biocompatibility was very similar to standard plastic used for cell culture, even though its surface structure differed from that of the plastic. Cells occupied comparable areas (68.9 ± 28.2% confluence for bSi vs. 63.3 ± 10.3% for plastic) and were spindle-shaped, with numerous processes. At the same time, bSi demonstrated enhanced cell proliferation: on bSi, the number of cells was almost two times larger (53.8 ± 15.2 cells per area for bSi vs. 36.0 ± 3.7 for plastic). Increased proliferation also led to an increase in cell growth density, reducing the area per each cell two times, as can be seen from Table 3.

Thus, rat C6 glioma cells, grown on the bSi as a substrate, demonstrated strong adhesion to the surface, a higher division rate than that obtained on the plastic of the tissue culture-treated dishes. Cells also preserved their usual morphology.

The PyC layer deposition on the bSi modified the adhesive properties of the surface: cell–surface contact became weaker, and as a consequence, the division rate decreased (Figure 4c). Cell confluence was only 20.7 ± 4.6% vs. 63.3 ± 10.3% in the control and 68.9 ± 28.2% on bSi. However, the surface remained biocompatible, cells preserved their usual morphology, were elongated, spindle-shaped, and with long processes. Their dimensional parameters also remained very similar to the control, even though the percentage of the area per each cell on the PyC on bSi was 80% of the percentage of the area per cell on the plastic (see Table 3).

A similar effect was observed for PyC of different thicknesses, deposited on flat quartz. Data are shown in Figure 4d,e and Table 3. For cells grown on PyC with a 20 nm thickness, the cell division rate was 2.3 times lower than in the control sample; cell confluence was 33.7 ± 7.1%, indicating lower proliferation. However, in the case of 40 nm PyC on SiO_2_ as a substrate, an increase in confluence and cell proliferation was observed, but still, it was lower than in the control sample. It is worth noting that neither 20 nm PyC nor 40 nm PyC modified the area per cell (%) parameter, and for rat C6 glioma cells, it remained approximately 2 for both PyC on SiO_2_ and the control samples. Moreover, the PyC layer on bSi increased this parameter from 1.3 ± 0.3 for bSi to 1.6 ± 0.1 for bSi with PyC, indicating that PyC does not modify cell morphology.

In the air, the bSi surface was covered by a 30–80 Å thick SiO_2_ layer. This oxide layer resulted in the positive surface charge at the interface, which improved the adhesion of cells to the surface because the outer surface of the cell membrane was negatively charged due to phosphate groups of the phospholipid bilayer. However, the deposition of highly conductive PyC led to a decrease in cell adhesion, as we observed for all our samples.

Vertical GNW on the Si wafer was associated with a further roughness decrease to nanoscale (thickness of the surface prominences oriented to the cell is defined by the graphene wall thickness). This led to a significant loss in cell adhesion and very poor biocompatibility, as shown in Figure 4e. Moreover, the distance between the GNW exceeded several microns. Cells lost long processes, did not form intercellular contacts, stayed round-shaped, and thus, lost the ability of effective cell division. This is the result of the high hydrophobicity of GNW [42]. Another effect reducing cell adhesion on GNW films may be related to the presence on their surface of needle-like species elongating above the flakes [23].

The cell–surface contact was provided by focal adhesions responsible for sensing environmental conditions. These specific protein complexes include transmembrane integrin molecules, associated with many cytoplasmic proteins regulating actin cytoskeleton dynamics, cell movement, and attachment [43]. Focal adhesions include “dot”-like or point contacts of 200–500 nm in the active edge of the cell, and elongated “dash” contacts of 2–10 µm in length and 0.5 µm in width, partially located under the nucleus [44]. These dimensional parameters of focal adhesion are critical in cell-surface contact formation as it strongly correlates with the interspike distance. Cell attachment is promoted by textures of low- and intermediate-roughness. It is inhibited by the increase in the interspike distance to 4.5 µm [45]. As seen from Figure 1, the distance between the surface prominences varied from approximately 70 nm for PyC on SiO_2_ (Figure 1c,i) to approximately 260 nm for bSi and PyC on bSi (Figure 1b,l). Under such conditions, the cells successfully spread over the substrate, as they could establish the next focal adhesion contact with surface prominences in any direction. At the same time, the thickness of GNW was less than the minimal required size of the focal adhesion contact of 200–500 nm. Therefore, it did not provide cells with the area for attachment, and the growth of the C6 glioma cells on such substrate was impeded. Thus, all fabricated surfaces except GNW met the requirements of dimensionality of surface texture roughness necessary for focal adhesions.

Poly-l-lysine, traditionally used for the improvement of cell adhesion to the growth surface, is a dielectric material, and like most biopolymers, has its own refractive index different from water, creates an uneven layer on the surface of the substrate, which significantly reduces the quality of signal transmission from the cell to the sensor electrodes, and degrades the quality of the optical images of cells introducing additional distortions into registered spectral characteristics of cells. PyC has significant advantages over the poly-l-lysine layer because this material can be directly used in the production of conductive–insulating surfaces for biosensors. Even though the PyC synthesis conditions require high temperature, which undoubtedly prohibits the use of classical optical glasses as a substrate, PyC can be successfully synthesized on quartz. It makes it possible to use these substrates with PyC for all types of NUV, visible, and NIR spectroscopies, as well as to transfer synthetized PyC to any other substrate, even flexible, for example, polydimethylsiloxane. In this case, any designed contact scheme can be created on the PyC by lithographic methods. All this becomes possible due to the unique electrical and optical properties of PyC, as well as the good biocompatibility and adhesive properties demonstrated in this study.

## 4. Conclusions

Special attention should be paid to macro-, micro- and nanoroughness of the materials that are intended to be used as functional substrates for biosensing/bioregulation platforms.

We demonstrate that GNW being roughly perpendicular to the Si substrate as well as PyC films of different thicknesses used in combination with microstructured surfaces (SiO_2_ and bSi) of different topography may provide both cell adhesion and proliferation promotion or arrest.

Rat C6 glioma cells strongly adhere to the bSi as a substrate. Cells are of spindle shape which corresponds to their usual morphology. The PyC layer on the bSi modifies the adhesive properties of the surface and smooths the bSi nanostructures. The cell–surface contact becomes weaker, and as a consequence, the division rate decreases. At the same time, the surface remains biocompatible (even without poly-l-lysine or other biopolymers commonly used for cell growth), providing the opportunity for the fine-tuning of cell growth speed.

To summarize, macro-, micro- and nanoscale roughness plays a crucial role in the process of cell adhesion to the biosensor surface. The combination of macroscopic and microscopic bSi roughness as well as surface charge distribution results in sufficient biocompatibility. In terms of adhesive properties, bSi can be compared to special plastics used for the cultivation of brain cells. BSi surface modification with PyC leads to decreased cell adhesion to the surface. This was observed both for PyC on bSi and PyC of different thicknesses on plain SiO_2_ substrates. Such PyC effect may be due to the fact that the PyC shields electric charges located on the surface and mimics nanoscale roughness. This will be used in the future for directed cell growth and intercellular contact formation for cells grown on 3D scaffolds covered with PyC. Further lowering the dimension of the surface roughness to nanoscale using vertical GNW-covered Si wafers demonstrates poor biocompatibility. The latter preparation can thus be used for the formation of superhydrophobic tracks for directed neuron network formation. The regulation of cell adhesion by changing roughness and the selective biocompatibility of investigated materials allow them to be applied further for the development of sensor platforms. The sensory properties of such platforms will be determined by the unique optical and electrical properties of the carbon-based materials.

## Figures and Tables

**Table 1:** Macroscopic and microscopic roughness of PyC (40 nm), PyC (20 nm) on SiO_2_, PyC (20 nm) on bSi and bSi (AFM images analysis).

Parameter	PyC (20 nm) on SiO_2_	PyC (40 nm) on SiO_2_	bSi	PyC (20 nm) on bSi
Surface Area Difference, %	0.3 ± 0.2	2.9 ± 2.5	136 ± 7	139 ± 9
Image R_q_, nm	0.9 ± 0.2	2.9 ± 1.3	81 ± 12	75 ± 9
Image R_max_, nm	11.2 ± 2.6	42 ± 19	499 ± 12	508 ± 37

Data are presented as the means ± CIs (*p* = 0.95, *n* = 10).

**Table 2:** Raman spectra characteristic modes of carbon multilayered materials deposited on different substrates.

Mode	PyC (40 nm) on SiO_2_	PyC (20 nm) on SiO_2_	PyC (20 nm) on bSi	GNWs on Flat Si
D mode, cm^−1^	1322.6 ± 2.1	1307.2 ± 1.3	1322.2 ± 2.2	1314.7 ± 3.1
G mode, cm^−1^	1595.2 ± 1.8	1602.1 ± 3.4	1595.2 ± 2.6	1580.8 ± 4.7

Data are presented as the means ± SDs of three independent measurements.

**Table 3:** Cell confluence and morphological features of cells grown onto different substrates.

Parameter	Plastic	PyC (20 nm) on SiO_2_	PyC (40 nm) on SiO_2_	bSi	PyC (20 nm) on bSi	Graphene Nanowalls
Area occupied by cells, %	63.3 ± 10.3	33.7 ± 7.1 *	55.1 ± 10.3	68.9 ± 28.2	^#^ 20.7 ± 4.6 *	11.6 ± 5.1 *
Area occupied by cells, µm^2^	25310 ± 4131	13475 ± 2848 *	22052 ± 4124	27570 ± 11261	^#^ 8271 ± 1855 *	4652 ± 2047 *
Average cell number per 200 × 200 µm area	36.0 ± 3.7	15.6 ± 1.1 *	25.2 ± 2.6 *	53.8 ± 15.2 ***	^##^ 13.0 ± 0.7 *	7.3 ± 0.4 *
Area per cell, %	1.8 ± 0.3	2.2 ± 0.1 **	2.2 ± 0.1 **	1.3 ± 0.3 ***	1.6 ± 0.1	1.6 ± 0.1
Area per cell, µm^2^	703 ± 115	864 ± 183 **	875 ± 164 **	513 ± 209 ***	636 ± 143	637 ± 280
Cell morphology characteristics	Elongated, spindle-shaped, 2–3 long processes, numerous intercellular contacts	Elongated, spindle-shaped, 2–3 long thin processes, few intercellular contacts	Elongated, spindle-shaped, 2–3 long thin processes, few intercellular contacts	Spindle-shaped, rounded, many middle-sized processes, many intercellular contacts	Elongated, spindle-shaped, 2–3 long thin processes, few intercellular contacts	Rounded, no processes or short wide processes, no intercellular contacts

Data are presented as the means ± SDs. The statistical significance of the results was analyzed by an unpaired *t*-test (* *p* < 0.001, ** *p* < 0.01, *** *p* < 0.05 vs. sample on plastic, ^#^ *p* < 0.05, ^##^ *p* < 0.001 vs. sample on bSi).

## References

[1] Dahinden C.A., Fehr J., Hugli T.E. (1983). Role of Cell Surface Contact in the Kinetics of Superoxide Production by Granulocytes. J. Clin. Investig..

[2] Spillmann C.M., Lomakina E., Waugh R.E. (2004). Neutrophil Adhesive Contact Dependence on Impingement Force. Biophys. J..

[3] Hoffman R., Benz E.J., Silberstein L.E., Heslop H.E., Weitz J.I., Anastasi J., Salama M.E., Abutalib S. (2017). Hematology: Basic Principles and Practice.

[4] Chen S., Lewallen M., Xie T. (2013). Adhesion in the Stem Cell Niche: Biological Roles and Regulation. Development (Cambridge). Development.

[5] Bian S. (2013). Cell Adhesion Molecules in Neural Stem Cell and Stem Cell—Based Therapy for Neural Disorders. Neural Stem Cells—New Perspectives.

[6] Akhmanova M., Osidak E., Domogatsky S., Rodin S., Domogatskaya A. (2015). Review Article Physical, Spatial, and Molecular Aspects of Extracellular Matrix of In Vivo Niches and Artificial Scaffolds Relevant to Stem Cells Research. Stem Cells Int..

[7] Bahrami S., Baheiraei N., Mohseni M., Razavi M., Ghaderi A., Azizi B., Rabiee N., Karimi M. (2019). Three-Dimensional Graphene Foam as a Conductive Scaffold for Cardiac Tissue Engineering. J. Biomater. Appl..

[8] Costa F., Carvalho I.F., Montelaro R.C., Gomes P., Martins M.C. (2011). Covalent immobilization of antimicrobial peptides (AMPs) onto biomaterial surfaces. Acta Biomater..

[9] Krutty J.D., Schmitt S.K., Gopalan P., Murphy W.L. (2016). Surface functionalization and dynamics of polymeric cell culture substrates. Curr. Opin. Biotechnol..

[10] Liao C., Li Y., Tjong S.C. (2018). Graphene Nanomaterials: Synthesis, Biocompatibility, and Cytotoxicity. Int. J. Mol. Sci..

[11] Jeong J.T., Choi M.K., Sim Y., Lim J.T., Kim G.S., Seong M.J., Hyung J.H., Kim K.S., Umar A., Lee S.K. (2016). Effect of Graphene Oxide Ratio on the Cell Adhesion and Growth Behavior on a Graphene Oxide-Coated Silicon Substrate. Sci. Rep..

[12] Syama S., Mohanan P.V. (2019). Comprehensive Application of Graphene: Emphasis on Biomedical Concerns. Nano-Micro Lett..

[13] Turon Teixidor G., Gorkin R.A., Tripathi P.P., Bisht G.S., Kulkarni M., Maiti T.K., Battacharyya T.K., Subramaniam J.R., Sharma A., Park B.Y. (2008). Carbon Microelectromechanical Systems as a Substratum for Cell Growth. Biomed. Mater..

[14] Das K., Madhusoodan A., Mili B., Kumar A., Saxena A.C., Kumar K., Sarkar M., Singh P., Srivastava S., Bag S. (2017). Functionalized Carbon Nanotubes as Suitable Scaffold Materials for Proliferation and Differentiation of Canine Mesenchymal Stem Cells. Int. J. Nanomed..

[15] Jin G.Z., Kim M., Shin U.S., Kim H.W. (2011). Neurite Outgrowth of Dorsal Root Ganglia Neurons Is Enhanced on Aligned Nanofibrous Biopolymer Scaffold with Carbon Nanotube Coating. Neurosci. Lett..

[16] Fabbro A., Bosi S., Ballerini L., Prato M. (2012). Carbon Nanotubes: Artificial Nanomaterials to Engineer Single Neurons and Neuronal Networks. ACS Chem. Neurosci..

[17] Bei H.P., Yang Y., Zhang Q., Tian Y., Luo X., Yang M., Zhao X. (2019). Graphene-Based Nanocomposites for Neural Tissue Engineering. Molecules.

[18] Menaa F., Abdelghani A., Menaa B. (2015). Graphene Nanomaterials as Biocompatible and Conductive Scaffolds for Stem Cells: Impact for Tissue Engineering and Regenerative Medicine. J. Tissue Eng. Regen. Med..

[19] Ryu S., Kim B.S. (2013). Culture of Neural Cells and Stem Cells on Graphene. Tissue Eng. Regen. Med..

[20] Li N., Zhang Q., Gao S., Song Q., Huang R., Wang L., Liu L., Dai J., Tang M., Cheng G. (2013). Three-Dimensional Graphene Foam as a Biocompatible and Conductive Scaffold for Neural Stem Cells. Sci. Rep..

[21] Mofazzal Jahromi M.A., Abdoli A., Rahmanian M., Bardania H., Bayandori M., Moosavi Basri S.M., Kalbasi A., Aref A.R., Karimi M., Hamblin M.R. (2019). Microfluidic Brain-on-a-Chip: Perspectives for Mimicking Neural System Disorders. Mol. Neurobiol..

[22] Lotti F., Ranieri F., Vadalà G., Zollo L., Di Pino G. (2017). Invasive Intraneural Interfaces: Foreign Body Reaction Issues. Frontiers in Neuroscience. Front. Media.

[23] Kleshch V.I., Vasilyeva E.A., Lyashenko S.A., Obronov I.V., Tyurnina A.V., Obraztsov A.N. (2011). Surface Structure and Field Emission Properties of Few-Layer Graphene Flakes. Phys. Status Solidi Basic Res..

[24] Kaplas T., Svirko Y. (2012). Direct Deposition of Semitransparent Conducting Pyrolytic Carbon Films. J. Nanophotonics.

[25] Hiramatsu M., Kondo H., Hori M. (2013). Graphene Nanowalls. New Progress on Graphene Research.

[26] Batrakov K., Kuzhir P., Maksimenko S., Paddubskaya A., Voronovich S. (2013). Enhanced Microwave Shielding Effectiveness of Ultrathin Pyrolytic Carbon Films. Appl. Phys. Lett..

[27] Cook S.D., Beckenbaugh R.D., Redondo J., Popich L.S., Klawitter J.J., Linscheid R.L. (1999). Long-Term Follow-up of Pyrolytic Carbon Metacarpophalangeal Implants. J. Bone Jt. Surg. Ser. A.

[28] Dovbeshko G.I., Romanyuk V.R., Pidgirnyi D.V., Cherepanov V.V., Andreev E.O., Levin V.M., Kuzhir P.P., Kaplas T., Svirko Y.P. (2015). Optical Properties of Pyrolytic Carbon Films Versus Graphite and Graphene. Nanoscale Res. Lett..

[29] Graham A.P., Schindler G., Duesberg G.S., Lutz T., Weber W. (2010). An Investigation of the Electrical Properties of Pyrolytic Carbon in Reduced Dimensions: Vias and Wires. J. Appl. Phys..

[30] McEvoy N., Peltekis N., Kumar S., Rezvani E., Nolan H., Keeley G.P., Blau W.J., Duesberg G.S. (2012). Synthesis and Analysis of Thin Conducting Pyrolytic Carbon Films. Carbon.

[31] Heikkinen J.J., Peltola E., Wester N., Koskinen J., Laurila T., Franssila S., Jokinen V. (2019). Fabrication of Micro- and Nanopillars from Pyrolytic Carbon and Tetrahedral Amorphous Carbon. Micromachines.

[32] Shah A., Stenberg P., Karvonen L., Ali R., Honkanen S., Lipsanen H., Peyghambarian N., Kuittinen M., Svirko Y., Kaplas T. (2016). Pyrolytic Carbon Coated Black Silicon. Sci. Rep..

[33] Ozturk S., Kayabasi E., Kucukdogan N., Ayakdas O. (2018). Progress in Applications of Black Silicon. Most Recent Stud. Sci. Art.

[34] Sainiemi L., Jokinen V., Shah A., Shpak M., Aura S., Suvanto P., Franssila S. (2011). Non-Reflecting Silicon and Polymer Surfaces by Plasma Etching and Replication. Adv. Mater..

[35] Hassler M. (2012). Other commonly used biomedical coatings: Pyrolytic carbon coatings. Coat. Biomed. Appl..

[36] Zhang W., Li A., Reznik B., Deutschmann O. (2013). Thermal expansion of pyrolytic carbon with various textures, ZAMM Zeitschrift Fur Angew. Math. Mech..

[37] Obraztsov A.N., Pavlovsky I.Y., Volkov A.P., Petrov A.S., Petrov V.I., Rakova E.V., Roddatis V.V. (1999). Electron Field Emission and Structural Properties of Carbon Chemically Vapor-Deposited Films. Diam. Relat. Mater..

[38] Magdesieva T.V., Shvets P.V., Nikitin O.M., Obraztsova E.A., Tuyakova F.T., Sergeyev V.G., Khokhlov A.R., Obraztsov A.N. (2016). Electrochemical characterization of mesoporous nanographite films. Carbon.

[39] Childres I., Jauregui L.A., Park W., Caoa H., Chena Y.P. (2013). Raman Spectroscopy of Graphene and Related Materials. New Developments in Photon and Materials Research.

[40] Baryshevsky V., Belous N., Gurinovich A., Gurnevich E., Kuzhir P., Maksimenko S., Molchanov P., Shuba M., Roddatis V., Kaplas T. (2015). Study of nanometric thin pyrolytic carbon films for explosive, electron emission cathode in high-voltage planar diode. Thin Solid Films.

[41] Lambin P. (2017). Graphene as a prototypical model for two-dimensional continuous mechanics. Appl. Sci..

[42] Samanta T., Biswas R., Banerjee S., Bagchi B. (2018). Study of Distance Dependence of Hydrophobic Force between Two Graphene-like Walls and a Signature of Pressure Induced Structure Formation in the Confined Water. J. Chem. Phys..

[43] Riveline D., Zamir E., Balaban N.Q., Schwarz U.S., Ishizaki T., Narumiya S., Kam Z., Geiger B., Bershadsky A.D. (2001). Focal Contacts as Mechanosensors: Externally Applied Local Mechanical Force Induces Growth of Focal Contacts by an MDia1-Dependent and ROCK-Independent Mechanism. J. Cell Biol..

[44] Owen G.R., Meredith D.O., Ap Gwynn I., Richards R.G., Bongrand P., Curtis A.S.G. (2005). Focal Adhesion Quantification—A New Assay of Material Biocompatibility?. Rev. Eur. Cells Mater. AO Res. Inst. Davos.

[45] Simitzi C., Stratakis E., Fotakis C., Athanassakis I., Ranella A. (2015). Microconical Silicon Structures Influence NGF-Induced PC12 Cell Morphology. J. Tissue Eng. Regen. Med..

